# Strength and failure mechanism of single-lap magnesium-basalt fiber metal laminate adhesively bonded joints: Experimental and numerical assessments

**DOI:** 10.1177/00219983221088095

**Published:** 2022-03-30

**Authors:** Fatemeh Mottaghian, Farid Taheri

**Affiliations:** Department of Mechanical Engineering, 152958 Dalhousie University, Halifax, NS, Canada

**Keywords:** Single-lap joint, Fiber metal laminate, Basalt fiber, Magnesium alloy, Surface treatment

## Abstract

A quick literature search reveals the significant lack of data and information concerning magnesium-to-magnesium bonded joints as well as fiber-metal laminates (FMLs) made with magnesium alloys. Therefore, a systematic series of experimental and numerical investigations are carried out to assess the performance of single-lap joints mating FML adherends. The primary goal is to better understand the effects of geometrical and material parameters that influence the performance of magnesium-to-magnesium joints. The FML adherends used in this study consist of basalt natural fiber-epoxy laminate sandwiched in between thin sheets of magnesium alloys, which were subsequently adhesively bonded using a room-cured epoxy resin. The effects of two types of surface treatments, namely, “sandblasting” and “sandblasting with resin coating” on the bond strength and failure mechanism of the adhesively bonded joints (ABJs) are investigated. A 3D numerical model developed to simulate the response of the joints subjected to quasi-static lap-shear tests. This model, which accounts for the material and geometrical nonlinearity in the joints, is used to perform a parametric analysis for establishing the optimal overlap bond length. The distributions of the shear and peel stresses in the overlap region and the effects of adhesive thickness on the performance of the joints are systematically examined. The comparison of the experimental data and numerical results confirms the robustness and cost-effectiveness of the numerical model in predicting the response of such single-lap ABJs.

## Introduction

Over the last few decades, energy conservation has become a major global priority due to growing fuel consumption, as well as increased emphasis on reducing Carbon dioxide (CO_2_) emissions. One of the most promising approaches for addressing the aforementioned issue is the greater usage of lightweight and low-cost materials including fiber-reinforced polymer (FRP) composites and fiber-metal laminates (FMLs) for developing lightweight components. FMLs were developed in the 90s as lightweight hybrid composite materials to take advantage of the ductility offered by metals and the positive attributes offered by FRPs. FMLs are therefore desirable alternatives to FRPs in that they compensate for the brittle response of FRPs, yet still offer lightweight, resilient, and cost-effective alternatives.

Lightweight materials have been widely adopted by several manufacturers to reduce fuel consumption in various transport vehicles.^1,2^ However, as the usage of such materials grows, the effective assembly of structural components produced by them, coupled with the maintenance of the required safety standards, have developed challenges for the industry. Besides the traditional joining procedures such as welding and mechanical fastening (e.g., bolting, riveting), adhesive bonding offers an effective method of joining, which is often opted in the industry such as aeronautics, space, and automotive industries.^3–5^ Offering a higher strength-to-weight ratio, enhanced stress distribution along the bond-line, relatively simplified fabrication procedures, improved corrosion and fatigue resistance, as well as the option of bonding dissimilar materials^6^ have made the application of adhesively bonded joints (ABJs) more attractive in joining many primary and secondary structural applications. However, to construct effective lightweight assemblies one must understand the response of the bonded joints mating the components. As stated, magnesium (Mg)-based FMLs are gaining an increasing popularity since they are even lighter than their traditional counterparts such as glass laminate aluminum reinforced epoxy (GLARE). However, there is a distinct lack of information and adequate database regarding bonded joints mating this class of super-lightweight FMLs. This paper attempts to provide information with the aim of narrowing the gap in the knowledge base.

In general, ABJs can be categorized into different groups based on their configurations, including the single-lap, double-lap, single-strap, double-strap, and scarf joints.^7^ Single-lap joints (SLJs) are the most often used configuration due to their standardization and ease of fabrication. In addition, SLJs have been experimentally and numerically investigated by numerous researchers,^8–11^ therefore are supported by a relatively large performance database. For instance, Anyfantis and Tsouvalis^12,13^ conducted a parametric study of seven different carbon-fiber-reinforced plastic (CFRP)-steel SLJ geometries subjected to lap-shear tests. They reported that the effects of the adhesive thickness and stiffness ratio were negligible on the performance of their joints compared to the effect of the overlap length. Zou et al.^14^ presented an elegant analytical model for assessing the performance of SLJs mating FRP. The model was further extended by Taheri and Zou^15^ to assess the performance of unsymmetric adhesively bonded composite sandwich panels-to-flange joints.

The complex stress distribution in the adhesive overlap region and the stress concentrations at the edges are the most common issues with SLJs affecting the strength of the joints. To improve and optimize the performance of SLJs, two major enhancement approaches, which are the adhesive material and configuration-related enhancements, have been suggested by various researchers.^16^ Durmuş and Akpinar^17^ examined the mechanical behavior of SLJ one-step lap joint (OSLJ) and three-step lap joint (TSLJ) with five different step lap lengths subjected to tensile loading numerically and experimentally. They reported that TLSJ carried the highest load among the considered types of joints, and the change in the step length in the TSLJ type led to a significant effect on the failure load of the joints. Jairaja and Narayana Naik^18^ studied performances of CFRP-Al SLJs prepared by using a single and a mixture of two adhesives (a brittle and a ductile). In which the Araldite-2015 ductile adhesive and the AV138 brittle adhesives were employed at the ends and in the middle of the bonded region, respectively. This study demonstrated that the use of dual adhesives led to higher bond strength.

Besides the enhancements obtained by modifying the adhesive material and joint configuration, a more effective surface preparation technique is also believed to significantly affect the adhesion and long-term performance of a given ABJ. Therefore, extensive research has thus been performed in order to boost the interface and enhance the bond strength of ABJs. Guo et al.,^19^ investigated the effects of different surface treatments including milling, sulfuric acid anodizing (SAA), phosphoric acid anodizing (PAA), and sandblasting with SAA on the bond strength of SLJ made of light-weight hard aluminum alloy subjected to tensile tests. They evaluated the improvement in the strength of SLJs as a function of their surface treatment and ranked them as PAA > Milling > SB + SAA > SAA. The effects of different surface treatments (e.g., manual sanding, grit blasting, and peel-ply plus grit blasting) on fatigue and tensile behavior of SLJs made of CFRP-laminates were analyzed by Park et al.^20^ The results of this study revealed that static strength retention at one million cycles was calculated at 55.4% for the sanding treatment, 47.5% for grit blasting, and 50.3% for peel-ply plus grit blasting. Xi et al.^21^ used two-step laser surface treatment to enhance the SLJ shear-lap performance of CFRP-laminates in which a 40.8% enhancement in shear strength of the specimens compared with untreated ones was reported. De Cicco and Taheri^22^ reported that using peel-ply with resin as a surface treatment in bonding magnesium sheets to FRPs led to the enhancement of mode I fracture toughness and delamination resistance under a low-velocity impact (LVI) loading state. A concise review of surface preparations techniques, including the application of atmospheric plasma surface treatments on the bond strength of ABJs are provided in the following reference^23^.

FMLs, which consist of FRP laminated composites interleaved among thin metallic sheets, provide synergistic advantages of metals and FRPs, including lightweight, ductility, superior impact properties, toughness and fatigue resistance. Consequently, they have been widely implemented in different industries in recent years as effective alternatives to metallic alloys.^24,25^ Since FML components would have to be joined in practice and that delamination is one of the common issues with FRPs and of FML performances, the investigation of the behavior of ABJs made with FML adherends under different loading conditions is of paramount importance. However, to date, to the best of the authors’ knowledge, there are very few studies on the ABJs mating FMLs. Bano et al.^26^ conducted research on SLJ carbon-reinforced Al laminates (CARALL) subjected to tensile loading state. They investigated the effect of different joint parameters such as the spews and stepped configuration, as well as incorporating nano-fillers reinforcement in their adhesive. The results revealed that the modified joints had better performance, and the addition of nano-fillers led to a higher joint strength. In another study, the mechanical responses of ABJs mating steel-based FMLs and CFRPs were studied by Lee and Song.^27^ They concluded that FML design had a significant role in increasing the adhesive strength and fatigue life.

As stated earlier, the lack of research on ABJs joining FMLs in general and joints mating Mg-based FMLs in particular, and the low volume of the currently available relevant database indicate that more investigation is required to better understand the response of FML-ABJs and enhance their performance under different loading conditions. Accordingly, the primary objective of this study is to evaluate the performance of magnesium-basalt (MB)-FML SLJs subjected to quasi-static tensile loading. As stated, this study was motivated due to the lack of research in joining this class of FMLs, especially the fact that Mg requires a completely different surface preparation process compared to other widely used metal counterparts (e.g., AI and steel). As mentioned earlier, surface treatment has a significant influence on the bond strength of ABJs, regardless of the strength of the adhesive selected for a given application. However, one of the issues encountered when using the conventional surface treatments technique on Mg alloys is the requirement for the immediate bonding of the treated surfaces. This may not be deemed practical in most industries. Furthermore, most of the available relevant studies have considered SLJs made of Al, steel and FRPs adherends; therefore, there is a clear need for characterizing the response of Mg-SLJ under various loading conditions.

It should be noted that compared to other commonly used metallic alloys, Mg alloys are lighter (e.g., 75% lighter than steel, 50% lighter than titanium, and 35% lighter than aluminum), consequently, the weight of Mg alloy structural components is comparable to that of FRPs. High strength-to-weight ratio and improved electromagnetic shielding capability are also among other advantages of Mg alloys. Based on a technical report of automobile mass-reduction technology delivered by Lutesy^28^ the cost of Mg alloys is approximately 20% higher than Al ones. However, despite the higher material costs associated with moving toward more mass-optimized metals and nonmetal, their potential for net component cost improvements leads to considering them as a promising alternative to advance their application in industries.

Cortés and Cantwell^29^ were among the first researchers who studied the fracture properties of Mg-CFRP-FML. They demonstrated that the tensile strengths of Mg-FMLs were higher than that of 2024-T0 Al-FMLs. They also concluded that the comparatively lower elastic modulus and fracture characteristics of Mg-FMLs could be diminished by choosing a suitable volume proportion of the FRPs. In another study,^30^ they also investigated the responses of FMLs made of Mg alloys, glass-FRP, and CFRP subjected to fatigue and LVI loading states and compared their performance against Al-FMLs. The highlight of this research was the indication of the superior corrosion resistance of Mg-FML, which appears to be contradictory compared to other investigators’ findings on the performance of Mg alloys in moist environments. Pärnänen et al.^31^ analyzed the LVI performance of Mg-FML in comparison with GLARE. The results indicated that the energy absorption capacity of Mg-FML was approximately equal to GLARE; however, the first-cracking load was considerably lower in the Mg-based FML. Vasumathi and Murali^32^ studied the behavior of jute/carbon Mg-FML and jute/carbon Al-FML subjected to tensile, flexure, and impact loadings conditions. Interestingly, the bending, tensile strengths, and stiffnesses of both FMLs found to be almost similar to the Al-FML by showing only between 10–15% superiority.

Moreover, there has been a significant thrust in recent years for the implementation of low-cost, eco-friendly, sustainable, and lightweight natural fibers as an alternative to conventionally used fibers such as glass, carbon, and aramid.^32^ The superior performance of basalt fiber-reinforced composites as a relatively low-cost and effective class of composite material has been demonstrated in.^33^ Besides their lower cost and superior mechanical properties, basalt fibers also offer several advantages such as being environmentally sustainable, excellent adhesion properties to resins, good resistance against fire and wear, and remarkable heat/sound insulation properties.^24,33^ The abovementioned facts further justify the necessity for research in joining such robust, effective and economical hybrid materials. Therefore, this research evaluates the effects of a series of important parameters that affect the performance and strength of single-lap ABJs mating MB-FMLs. The parameters investigated include (i) two types of surface treatments, (ii) overlap length, and (iii) adhesive layer thickness. The failure mechanism of the SLJs is investigated by utilizing the “sandblasting” and “sandblasting with resin coating” surface treatment methods. Moreover, an FE model is developed in the LS-DYNA environment by which the variations in the stress distributions in the overlap region in SLJs and bond strength of SLJs with different overlap lengths and adhesive thicknesses are systematically evaluated and compared. The numerical framework accounts for the material and geometrical nonlinearities.

## Experimental investigation

### Materials

The MB-FML consists of Mg alloy sheets, basalt fabrics, and a room-cured epoxy resin. AZ31B–H24 Mg sheets with 0.5 mm thickness were purchased from MetalMart (Commerce, CA, US). The (0/90) bidirectional stitched basalt fabric with a thickness of 0.55 mm and an areal density of 450 g/m^2^ was obtained from the GBF basalt fiber Co., Ltd (China). Moreover, the room-cured structural epoxy system (West System 105 resin and 206 slow hardener) was acquired locally.

### Fabrication procedure and configuration of the joints

#### MB-FML fabrication

MB-FML Specimen preparation began with the fabrication of its FRP core constituent by preparing the basalt-epoxy laminate panels using the vacuum-assisted resin infusion (VARI) technique.^34^ The reinforcing basalt fabrics with [0/90]_2S_ layup were covered with peel-plies and resin distribution mesh. The system was sealed in a vacuum bag and monitored for a leakage test for 20 min. Then, the room-cured epoxy resin and hardener were mixed with a weight ratio of 5:1 and infused into the dry fabrics, and let cure at ambient temperature under vacuum for 48 h. In the next step, the cured basalt-epoxy laminate panels were sandwiched in between two pre-treated Mg sheets, which will be thoroughly described in *Surface Treatment*, using the same resin system, which was brushed onto the mating surfaces. Subsequently, the assembly was sealed by the vacuum bagging process and allowed to cure at ambient temperature for 48 h, as per the manufacturer’s recommendation.

### SLJs fabrication

**Figure 1.**
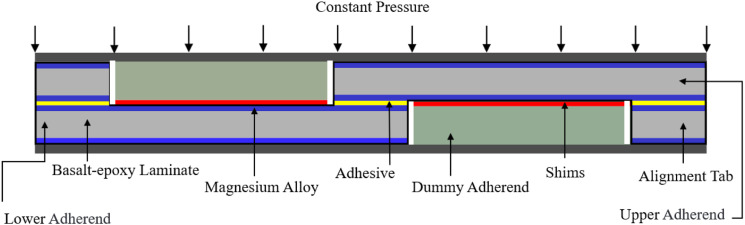
Schematic of the fabrication scheme used to produce the single-lap MB-FML ABJs.

**Figure 2.**
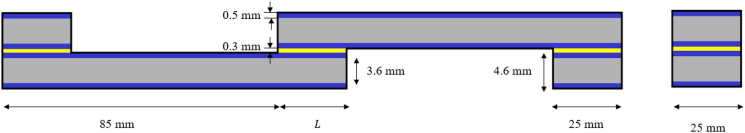
Schematic and dimensions of single-lap MB-FML ABJs.

Fabrication of SLJs is commenced by extracting the appropriate size upper and lower substrate plates from the mother MB-FML panels. Since as stated the thickness of adhesive in the overlap bonded regions plays an important role in the performance of SLJs, special adjustable fabrication jigs and procedures were adopted to facilitate the fabrication process and to ensure consistency and geometric precision of the fabricated SLJs. The schematic shown in Figure 1 provides a visual perspective of the fabrication setup. The area of the overlap was marked, and then the mating regions were wiped clean with acetone and left to fully airdry. To control the desired adhesive thickness, dummy adherends were prepared and placed below the upper adherend as well as on top of the lower adherend in the joint assembly, and then shims were positioned over and under the dummy adherends, respectively. The room-cured adhesive was brushed onto the bonding regions and the upper and lower adherends were bonded together. Subsequently, to eliminate the eccentricity in the loading path, each adherend was bonded to its corresponding alignment tab by the room-cured epoxy resin. In the next step, constant pressure was applied to the upper surface of the assembly area to ensure the appropriate bonding, as per the manufacturer’s recommendation. The resulting excess adhesive seepage was cleaned by a slender wooden stick to finalize the joints with 90° fillets. Afterward, the entire system was allowed to cure at ambient temperature for 48 h. Next, the SLJ specimens, with appropriate dimensions as per based on ASTM D5868-01 and ASTM D1002-10,^35,36^ were extracted from the bonded plates using a water-cooled diamond saw (see Figure 2).

### Surface treatment

As mentioned previously, surface preparation plays a significant role in the bond strength of ABJs. Therefore, two surface treatment approaches (namely “sandblasting” and “sandblasting with resin coating”^22^) are explored in this study. The two approaches are referred to as the “S” and hybrid “H” methods, hereafter.

The “S” approach commenced with cleaning the Mg sheets with acetone to remove any residual protective grease. The Mg sheets were subsequently sandblasted with 20–30 grit crushed glass to roughen the surfaces and promote better adhesion of the adhesive. Incidentally, both surfaces of each Mg sheet had to be sandblasted to avoid any resulting residual stress and slight curvature that would be resulted otherwise if only one of the surfaces in such thin sheets is sandblasted. Next, the sandblasted sheets were cleaned with compressed air and then wiped with acetone to remove any potential impurities and residues left from sandblasting.

In the “H” surface modification approach, after carrying out the above procedure, a thin layer of the room-cured resin was applied on the surfaces of the Mg sheets. Subsequently, the coated surfaces were covered with a layer of porous peel-ply and breather cloth. The thicknesses of the nylon peel-ply and the polyester breather cloth are 0.1016 and 3 mm, respectively. The assembly was then placed inside a vacuum bag left to cure at the ambient temperature for 48 h. It should be noted that in this method, the peel ply leaves a rough resin impression on the coated surface, thereby promoting a more effective adhesion.^22^ The other advantages of this approach which is also very elaborate and complex surface preparation method have been detailed in.^22^ It should be noted that as stated, the effectiveness of the hybrid surface treatment in reference to Mg-to-glass epoxy bonding was examined in one of earlier studies^22^; however, the integrity of this technique would have to be examined and verified for Mg-to-Mg bonding, which is one of the objectives of the present work.

### Test setup

**Figure 3.**
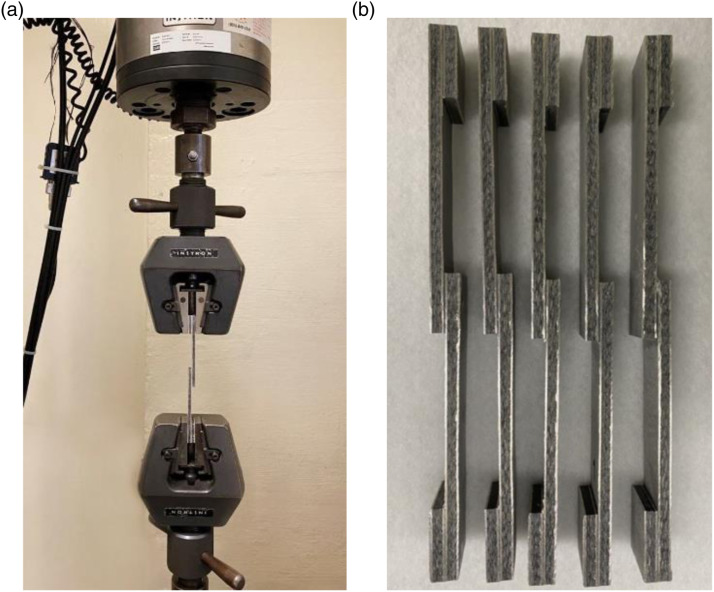
(a) lap-shear test set up, (b) MB-FML SLJ specimens.

In this study, the quasi-static single-lap shear tests were conducted using an MTS servo-hydraulic universal testing machine equipped with a FlexTest-40 controller and a 250 kN Instron load cell. To have accurate experimental results, based on Figure 2, at least five specimens were organized in each group, which were tested at a constant crosshead displacement rate of 1.27 mm/min as per ASTM D1002-10.^35^ The quasi-static shear-lap setup and the single-lap Mg-FML specimens are illustrated in Figure 3.

## Results and discussion

### Experimental results

#### Effects of surface treatment on the bond strength of SLJs

As mentioned previously, two types of surface treatments were considered to prepare the surfaces of the metal sheets for bonding with the aim of attaining more effective bonding conditions. The specifics of the surface preparations methods were outlined in *Surface treatment*. Identification and compositions of the groups of single-lap MB-FML ABJs fabricated in this study are reported in Table 1. The first two parts of the specimen IDs are self-explanatory; the third part refers to the adopted surface treatment method (i.e., (S) for sandblasting and (H) for hybrid) and the last numerical part denotes the overlap length of the joint in the unit of mm.

**Table 1.** Categories of the fabricated single-lap MB-FML ABJ specimens.

Samples ID	Surface treatment	Overlap length (mm)	Failure mode
MB-FML-S-25	Sandblasting	25	Adhesion
MB-FML-H-25	Sandblasting + Resin coating	25	Adhesion + Cohesion
MB-FML-H-20	Sandblasting + Resin coating	20	Adhesion + Cohesion
MB-FML-H-35	Sandblasting + Resin coating	35	Adhesion + Cohesion

**Figure 4.**
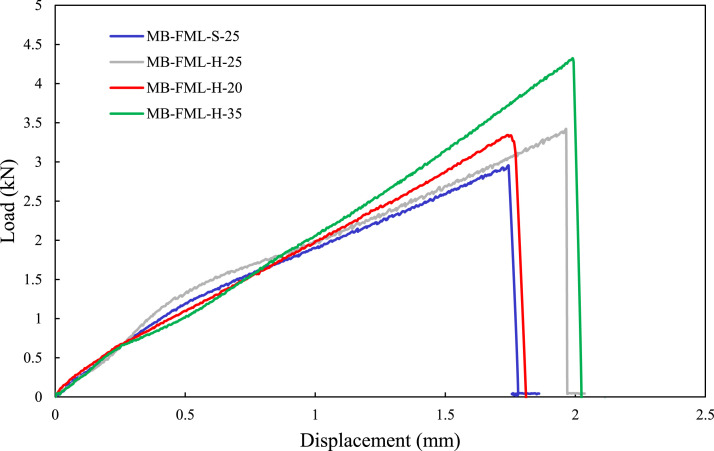
Load-displacement responses of the single-lap MB-FML ABJs.

**Figure 5.**
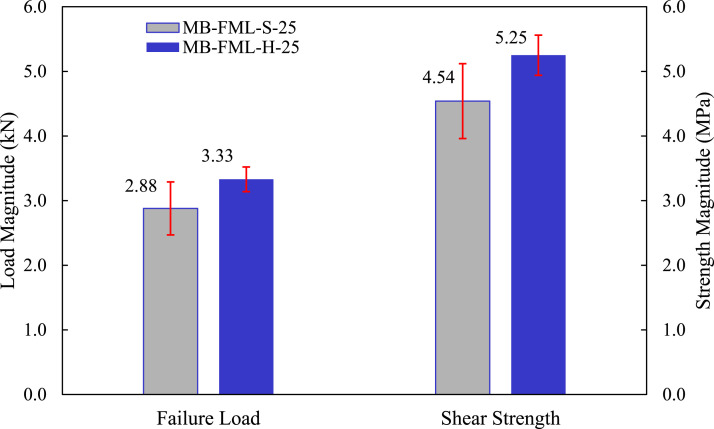
Experimentally obtained average failure load and shear strength of MB-FML-S-25 and MB-FML-H-25 group of specimens.

The load-displacement responses of the group of specimens subjected to the single-lap shear tests are exhibited in Figure 4. The average failure loads of MB-FML-S-25 and MB-FML-H-25 groups were determined to be 2.88 and 3.33 kN, respectively, rendering a 15.6% gain in the ultimate capacity in joints prepared with the hybrid surface treatment. Note that specimens with the overlap of 25 mm in Figure 4 were fabricated based on ASTM D5868-01 and ASTM D1002-10^35,36^ and the specimens with the overlap of 20 mm and 35 mm were fabricated based on the numerical results carried out in this study, which will be thoroughly discussed in *Optimized Overlap Length*. The average shear strength of the specimens was obtained using the simple mechanics of materials approach with the following equation

(1) $$\tau =\frac{F}{L\times w}$$

where F , τ , w ,  L denote failure load, average shear strength, joint width, and the overlap length, respectively. Figure 5 shows the average failure loads and the maximum shear stresses of the single-lap MB-FML ABJs fabricated with sandblasting and hybrid surface treatments and their standard deviations. As it can be seen, the hybrid surface treatment improved the average shear strength of bonded joints by 15.7%. When adhesive is applied on treated surfaces, the adhesive fills the cavities that were created on the surfaces, forming finger-like connections on the irregular topography created on the adherend surfaces once the adhesive is cured.^37^ This phenomenon, described as mechanical interlocking, is primarily affected by the roughness and porosity of the treated surfaces. Based on the results illustrated in Figure 4 and Figure 5, it can be concluded that the generated mechanical interlocking worked more effectively in the SLJs group of specimens fabricated with the hybrid (H) surface preparation technique. This technique is believed to generate greater surface roughness, thus, enhancing the strength of the single-lap MB-FML ABJs. This adopted method is simple and cost-effective, and in contrast to the conventional sandblasting method; it would also inhibit the oxidation and corrosion of Mg sheets. Consequently, there would be no urgency to immediately follow the surface preparation with the bonding process, which would be necessary otherwise. Therefore, this flexible surface preparation method was adopted for fabricating the remaining ABJs used in this study.

#### The failure mechanism of SLJs

**Figure 6.**
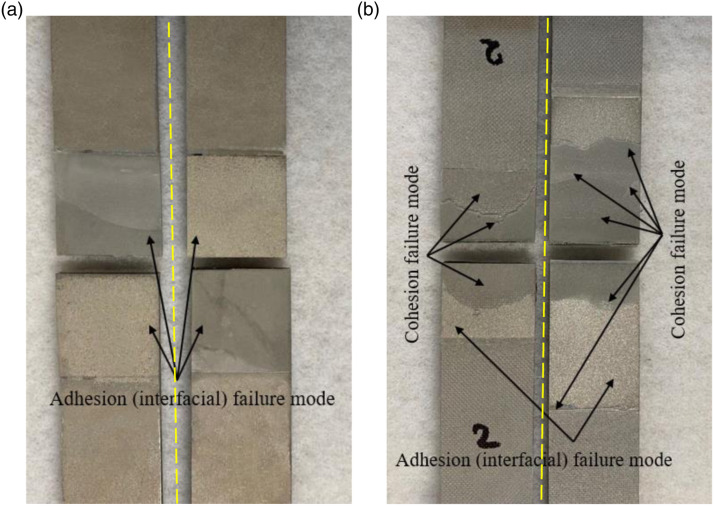
The fracture surfaces of the single-lap MB-FML ABJs (a) adhesion, (b) cohesion failure modes.

The failure mode of a given ABJ is considered to be an indicator of the adhesion quality and joint strength. In general, failure modes in ABJs are classified into four categories. First is the cohesion mode, which is the most desired failure mode in which the failure occurs in the layer of the adhesive itself. The next mode, or the adhesion (or interfacial) mode, signifies the failure of the adherend/adhesive interface. This type of failure is believed to occur due to inadequate surface treatment and lack of interlocking in between the adherend and adhesive. There would also be the mixed cohesion/adhesion mode, which often occurs when the overlap region experiences a relatively large rotation (due to the developed bending moment). Finally, there would be the adherend mode, which is characterized by failure of the adherend(s) instead of the adhesive.^38^ Since the adherends are offset in SLJs and the force path is nonconcentric, a bending moment is developed consequently.^39^ This could also occur due to the relative flexibility of the overlap region in such slender systems when tabs are used to minimize the load.^18^ The resulting overlap rotation due to the developed bending moment is a function of the adherends and adhesive thickness, the distance between the alignment adherends and overlap region, leading to the development of peel and shear stress concentrations near the ends of the overlap region.^40^ Consequently, the damage is usually initiated in the ends of the overlap region, subsequently propagating, and causing the failure of SLJs. As seen in Figure 6(a), in MB-FML-S-25, the crack developed at the end of the overlap region and then propagated toward the mid-span of the region. This crack propagation subsequently resulted in debonding of the adhesive/Mg alloy interface. As seen in the figure, a layer of adhesive remains on one of the bonding surfaces of one of the adherends. Nevertheless, the mixed cohesion/adhesion mode exhibited in Figure 6(b) was the predominant failure mode in the overlap region of the remaining ABJs tested in this study. Conforming to the self-explanatory failure mode’s name, thin patches of adhesive can be seen on both metal interfaces, indicating the failure plane passed through the adhesive in that region. However, the other portion of the overlap region experienced decohesion of the adhesive from one of the adherends. As can also be discerned, there is no rough resin impression on the failure adhesive regions, indicating the failure did not occur between the coating and adhesive, hence, it is a cohesion failure. The transition from the adhesion (or interfacial) mode of failure to the mixed cohesion/adhesion debonding mode indicates that the hybrid method of surface treatment (e.g., sandblasting with resin coating) was more effective compared to sandblasting in boosting the surface roughness and improving the bond strength. A summary of the surface treatments and failure modes of different groups of specimens is presented in Table 1

### Numerical investigation and results

**Figure 7.**
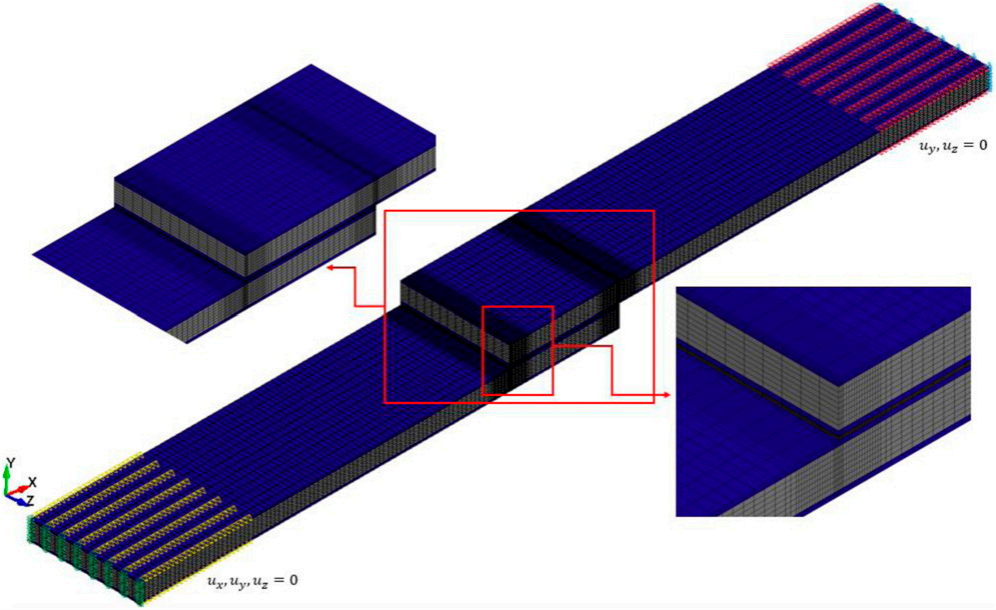
Details of the FE model of the single-lap MB-FML ABJ and the boundary conditions.

In this study, the commercial FE software LS-DYNA was utilized to model the response of the single-lap MB-FML ABJs subjected to the quasi-static tensile loading state. Constructing a reliable and robust numerical model can assist one to predict the performance of the ABJs subjected to different loading conditions and facilitate the analysis of complex stress distribution within the adhesive layer. An implicit nonlinear FE analysis was conducted to establish the joint’s behavior, accounting for both the geometrical and material nonlinearities of the joints. The model consists of three components (i) the Mg alloy sheets, (ii) the basalt-epoxy laminate layers, and (iii) the overlap adhesive layers. Figure 7 illustrates the single-lap MB-FML ABJs, in which all the constituents are meshed with the eight-node hexahedron fully integrated solid element (ELFORM = 2) of LS-DYNA. Coarser mesh densities were adopted to model the portions of the adherends away from the overlap region, especially near the ends of the region where stress gradients rapidly change. In other words, the mesh density was gradually refined as one nears the edges of the overlap region, which are necessary to capture the rapidly changing stress gradients and produce accurate numerical results. To mimic the boundary conditions in the gripped regions (within 25 mm at either end), all degrees of freedom (DOF) of the nodes located at one gripped end of the specimens were fully restrained. On the other gripped end, all the degrees of freedoms of the nodes except those corresponding to the axial movement ( $u_{x}$ ) were also restrained. The displacement-controlled algorithm was employed to simulate the experimentally imposed axial quasi-static movement (loading) in this nonlinear implicit analysis.

**Figure 8.**
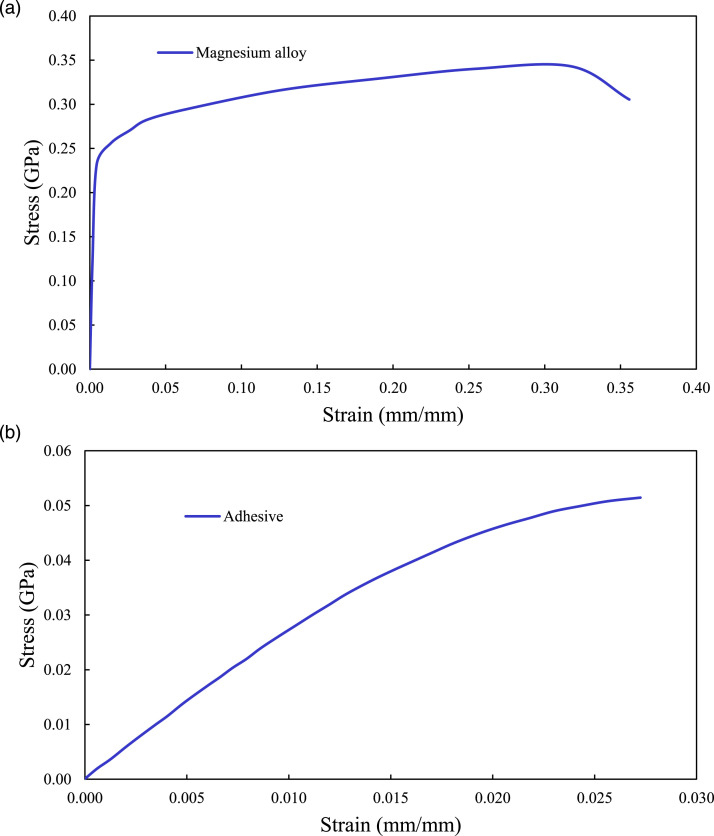
Stress-strain responses of (a) Mg alloy, (b) adhesive utilized in the FE model.

As for modeling the materials’ responses, the Mg alloy sheets were modeled as a piece-wise plastic material by considering both the strain-rate effect and yield stress ( $\sigma_{y}$ ) of the material. The basalt-epoxy laminate was modeled as a linear orthotropic material, and the adhesive layer was modeled also as a plastic material using the polymer plasticity model of the code. The nonlinear responses of both materials were modeled using their actual stress-strain responses. Note that the polymer plasticity model used in LS-DYNA for modeling adhesive layer is capable of simulating materials that do not exhibit a distinct change from elastic to plastic responses in their stress-strain curve, whereas the piecewise model could contain a linear-elastic region. The mechanical properties, as well as the material keywords utilized in LS-DYNA to simulate the constituents of single-lap MB-FML ABJs are tabulated in Table 2 and Table 3, respectively. Also, the stress-strain responses of Mg alloy and adhesive utilized in the FE model are illustrated in Figure 8.

**Table 2.** Mechanical properties of the materials used in the FE model.

Property	Thickness (mm)	ρ $\left(\mathrm{Kg}/m^{3}\right)$	$E_{11}$ (GPa)	$E_{22}$ (GPa)	$v_{12}$	$v_{13}$	$v_{23}$	$G_{12}$ (GPa)	$G_{13}$ (GPa)	$G_{23}$ (GPa)	$\sigma_{y}$ (GPa)	$\sigma_{u}$ (GPa)
Basalt-epoxy^41^	0.45	1860	37.95	9.82	0.3	0.3	0.19	2.80	2.80	4.14	—	—
Mg alloy^42^	0.50	1740	36.00	—	—						0.23	—
Adhesive^43^	0.30	1180	3.17	—							—	0.05

**Table 3.** LS-DYNA Material models utilized in constructing the FE model.

Constituent	Material model
Mg alloy	*PIECEWISE_LINEAR_PLASTICITY (MAT_024)
Basalt-epoxy	*MAT_ORTHOTROPIC_ELASTIC (MAT_002)
Adhesive	*MAT_PLASTICITY_POLYMER (MAT_089)

**Figure 9.**
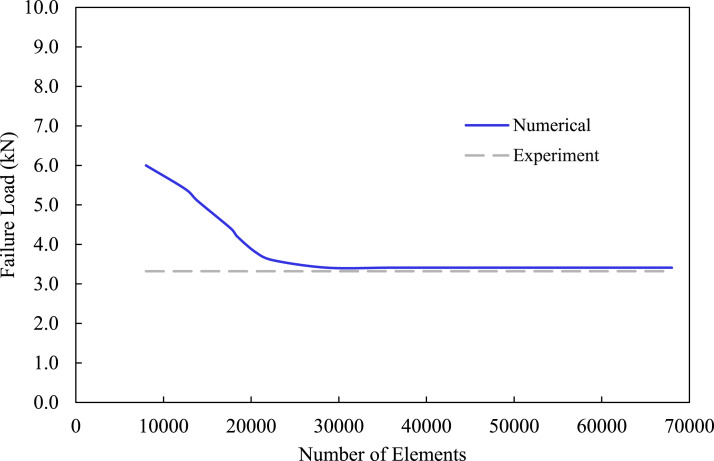
Convergence analysis of FE model conducted using MB-FML-H-25 ABJs.

In the first step of the numerical analysis, a mesh convergence study was conducted on the MB-FML-H-25 group of specimens to establish the integrity of the modeling framework. The number of elements in the overlap region, through-the-thickness of FML and adhesive interface layer, and within the plane of the FE model were changed successively. Moreover, the ultimate capacity of the joint was selected as the convergency criterion by comparing the FE-produced capacity with that evaluated experimentally. Consequently, as exhibited in Figure 9, the optimized mesh configuration is achieved by 28,630 solid elements with 35,072 nodes beyond which no significant improvement in accuracy could be obtained. A comparison between the numerical results of SLJs modeled with and without consideration of the tabs was carried out. The difference in the failure load in both linear and nonlinear analyses was found to be 1.04% and 0.75%, respectively. It was also found that the same magnitude of bending moment was generated in the models with and without tabs. Therefore, for the sake of computational efficiency, the model without tabs was considered in this study as opted by other researchers as well.^17,18^ The ultimate strength of joints in this study refers to the resulting strength at the stage when the maximum shear stress in the overlap region exceeds the ultimate shear strength of the adhesive. The numerically obtained failure load for MB-FML-H-25 SLJ is reported as 3.41 kN. The good agreement in the numerical and experimental results and the associated low error margin of 2.4% confirms the integrity of the FE model.

#### Optimized overlap length

**Figure 10.**
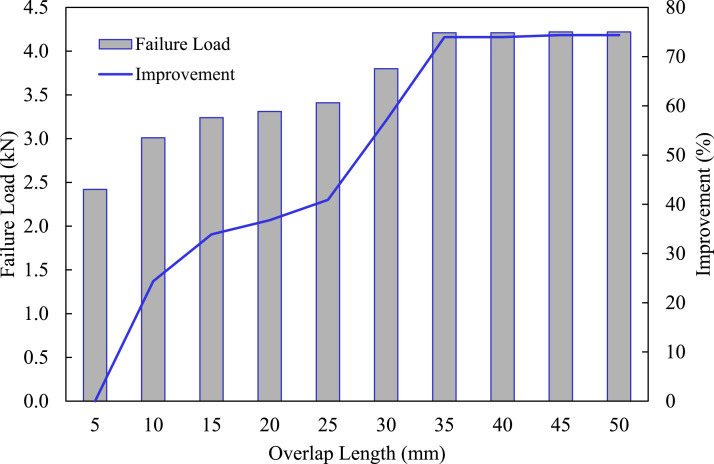
Effects of overlap length on the failure load of single-lap MB-FML ABJs.

As stated earlier, an ABJ’s performance is affected by parameters such as the thickness and stiffness of its adhesive and adherends, overlap length, as well as spew fillet angle. Among all the aforementioned parameters, the overlap length is believed to have a significant influence on the performance and bond strength.^41^ However, a longer overlap length does not translate into more capacity. Therefore, a parametric study was conducted to numerically establish the optimal performance of the single-lap MB-FML ABJs based on the resulting joint capacity. For that, 10 different overlap lengths were considered as the initial parameter. The ultimate load capacity produced by the different lengths is illustrated in Figure 10. The results indicate that an overlap length of 35 mm would produce the optimal joint capacity beyond which no significant gain in capacity would be obtained. The transition in the overlap length from 35 mm to 5 mm would result in a 74% decrease in the joint capacity.

**Figure 11.**
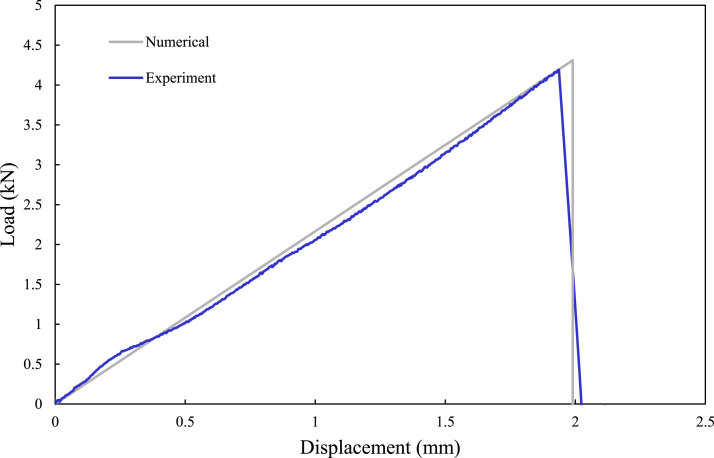
Comparison of the numerical and experimental load-displacement responses of MB-FML-H-35 group of specimens.

To further explore the integrity of the developed FE framework, a joint with a mid-range overlap length of 20 mm (i.e., MB-FML-H-20) was fabricated and tested. The joint’s performance was compared against the other joints, including the joint with the optimal overlap length of 35 mm (i.e., MB-FML-H-35). The experimentally obtained load-displacement responses of the two joints are shown in Figure 4. As can be seen, the capacity of the MB-FML-H-35 is 29.7% and 25.8% higher than the capacities of MB-FML-H-20 and MB-FML-H-25, respectively. Furthermore, the experimental and numerical joint capacities of the referenced ABJs are also tabulated in Table 4. One can see that the results produced by the developed FE model are in excellent agreement with the numerical results with low error margins varying between 1.4% for MB-FML-H-35 to 2.5% for MB-FML-H-20 groups of specimens. Therefore, the FE model is capable of effectively predicting the response of the MB-FML SLJs. Also, the numerical load-displacement response of the joint with the optimal overlap length (i.e., MB-FML-H-35) is compared to the experimental data and shown in Figure 11, which further reveals the integrity of the developed FE models.

**Table 4.** Comparison of the experimental and numerical failure loads of single-lap MB-FML ABJs.

Specimens ID	Overlap length (mm)	Exp. Failure load (kN)	Num. Failure load (kN)	Failure load ratio (Exp/Num)	Error (%)
MB-FML-H-20	20	3.23	3.31	0.976	2.5
MB-FML-H-25	25	3.33	3.41	0.977	2.4
MB-FML-H-35	35	4.19	4.25	0.986	1.4

#### Stress distribution in the adhesive

**Figure 12.**
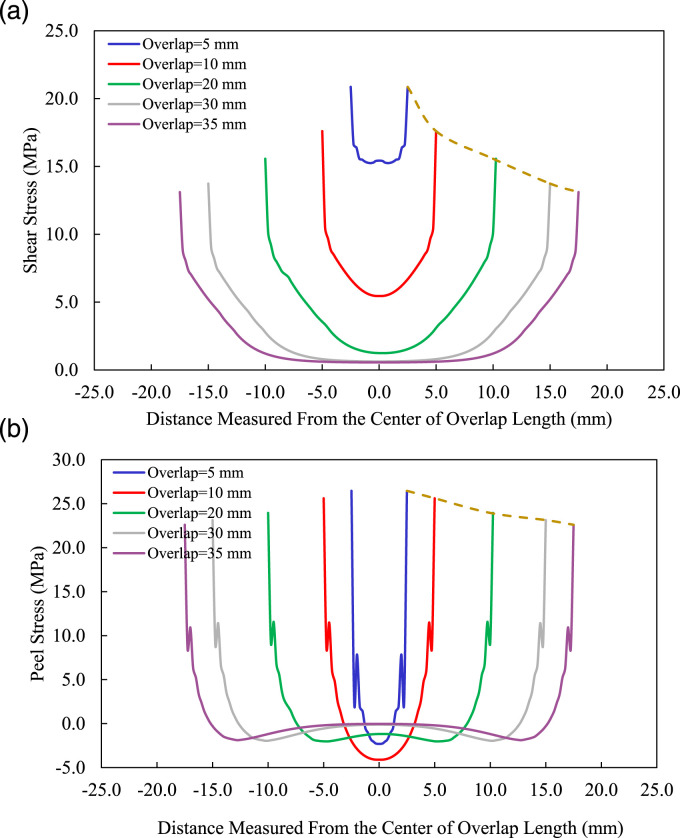
Effects of overlap length on the distribution of (a) shear and (b) peel stresses.

As briefly stated earlier, the resulting nonlinear response of SLJ results in the development of a bending moment in the overlap region and consequently leads to the development of the peel and shear stresses. The stresses in the ABJs are transferred from one adherend to the other one through the adhesive layer. Therefore, to gain a better understanding of the performance of single-lap MB-FML ABJs subjected to a tensile loading state, a more detailed stress analysis of the adhesive layer is carried out in this section. Figure 12 demonstrates the adhesive peel ( $\sigma_{y}$ ) and shear ( $\tau_{xy}$ ) stress distributions along the overlap region in joints with different overlap lengths. Note that the stress distribution is evaluated under an applied tensile load of 2 kN for all joints. As can be seen, both the peel and shear stress distributions are symmetrical about the overlaps’ midspan, and their magnitudes are maximized at the free edges of the adhesive layers, particularly where a crack(s) would be initiated and propagated.^13^ By increasing the overlap length, the peel and shear stresses’ peaks decrease and a relatively more uniform stress distribution along the overlap is achieved. In joints with longer overlap lengths, the stresses in the middle of the overlap region are relatively small. In other words, in the optimized group of specimens (MB-FML-H-35) a relatively long and stable plateau is obtained which separates the stress distribution at the free edges and middle of the overlap length.^42^ Clearly, the elongation of the overlap region beyond a certain length results in the reduction of both shear and peel stresses concentrations at the free edges and consequently leads to the enhancement of the ultimate load-bearing capacity.^43^ However, the rate of reduction in the stresses is not constant and it essentially changes slightly in the joints with longer overlap lengths, indicating that from an economical perspective, the bond length should be elongated to a certain length. This phenomenon is also supported by the results discussed in the previous section. Moreover, overlap length has a more noticeable effect on the shear stress (its concentration at near the free edges) in comparison to its effect on the peel stress. The relatively lower and negative values of peel stress, which denotes the existence of normal compressive stress (which in favorable this case), are observed along a large portion of the overlap region.^44^

**Figure 13.**
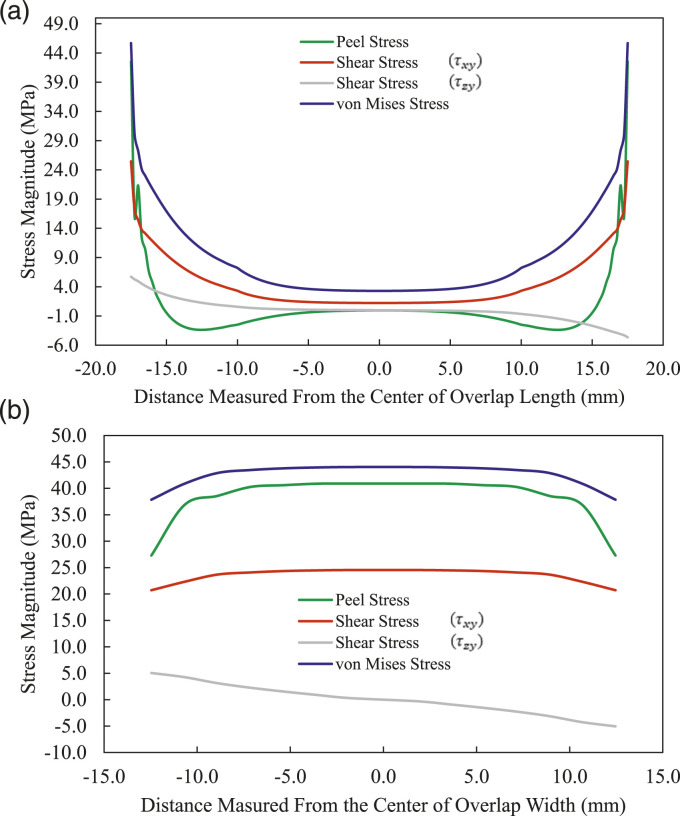
Stress distributions (a) along the length, (b) across the width of the overlap in MB-FML-H-35 group of specimens.

The distributions of the peel, and shear ( $\tau_{xy}\;\text{and}\;\tau_{zy}$ ) and von Mises ( $\sigma_{V}$ ) stresses along the overlap length and across the width of the adhesive layer in the MB-FML-H-35 specimen are presented in Figure 13. Note that the stresses are sampled at the maximum load of the mentioned joint. As can be seen, with the exception of shear stress ( $\tau_{zy}$ ) distribution, which is asymmetrically distributed, all the other stresses are symmetrically distributed with respect to longitudinal and transverse directions. The asymmetric distribution is due to the asymmetrical nature of SLJs geometry. Furthermore, the maximum shear stress ( $\tau_{xy}$ ) exhibited in Figure 13(a) is equal to the ultimate shear strength of the adhesive (i.e., 25 MPa); however, the peel stress remains significantly lower than the adhesive’s ultimate tensile strength of 50 MPa. This implies that the high shear stress developed at the free edges of the overlap region would initiate the failure of the joint; consequently, the SLJ failure mode is shear-governed. The magnitude of shear stress ( $\tau_{zy}$ ) remains at very low levels both along the length and across the width of the overlap and stays just in the elastic region; therefore, it does not contribute to the failure of SLJs significantly. Based on the results presented in Figure 13(b), it can be concluded that the peak magnitudes of the peel, shear ( $\tau_{xy}$ ), and von Mises stress, are reached across the width in the region slightly away from the edges, diminishing in regions close to width-free edges. However, in the case of shear stress ( $\tau_{zy}$ ), its maximum and minimum magnitudes occur near the left and the right free edge across the overlap width, respectively, nullifying in the mid-width of the region. As can be seen, the differences between the maximum and minimum values of three of the four illustrated stresses are not significant.

#### Effects of adhesive thickness on the bond strength of SLJs

Adhesive thickness is considered as one of the primary parameters governing the response of SLJs. To further understand the response of single-lap MB-FML ABJs subjected to a tensile loading state, an attempt was made to numerically assess the effects of adhesive layer thicknesses on the load-bearing capacity of the SLJ. For this, the maximum load, as well as the ultimate shear strength (based on equation (1)) of SLJs with the overlap length of 35 mm, are assessed and reported in Table 5. As can be seen, by increasing the adhesive thickness from 0.1 mm to 0.4 mm, the ultimate capacity of the joints increases from 2.91 kN to 4.56 kN, respectively. In other words, the failure load of an MB-FML SLJ with a thickness of 0.4 mm would be 56.7% higher than that of the joint with 0.1 mm thick adhesive. The incremental increase in the adhesive thickness in the selected range results in decreasing the shear stress concentration, thereby leading to a stronger joint. However, there is a threshold for the thickness after which no improvement in strength would result by increasing the adhesive’s thickness. Indeed, the increase in the thickness beyond the threshold reduced the load-bearing capacity slightly (by 1.1% and 2.2% corresponding to the thicknesses of 0.5 mm and 0.6 mm, respectively, in comparison to the joint with a thickness of 0.4 mm). The phenomenon corroborates with the observation made by other researchers^45^ who demonstrated optimal adhesive thickness of 0.5 mm of their investigated SLJ, beyond which the load-bearing capacity of SLJ was decreased. Moreover, the maximum shear strength in the joint with the optimal adhesive thickness is 5.21 MPa, revealing a 56.7% improvement in comparison to the base group of specimens (e.g., the SLJ with 0.1 mm thickness). After this step, further increases in the adhesive thicknesses result in diminishing the shear strength.

**Table 5.** Effects of adhesive thickness on the failure load and shear strength of single-lap MB-FML ABJs with 35 mm overlap.

Adhesive thickness (mm)	Num. Failure load (kN)	Num. Shear strength (MPa)	Improvement (%)
0.1	2.91	3.33	0.0
0.2	3.47	3.97	19.2
0.3	4.21	4.81	44.7
0.4	4.56	5.21	56.7
0.5	4.51	5.15	55.0
0.6	4.46	5.10	53.3

## Summary and conclusion

This study was initiated in response to the clear lack of information on the adhesively bonded joints with a Mg-to-Mg interface. In this study, a series of systematic experimental and numerical analyses were carried out to investigate the performance of a single-lap adhesively bonded joint made of fiber-metal laminates consisting of basalt-epoxy laminate and Mg alloy (MB-FML ABJs). The combination of the materials results in the formation of significantly lightweight and cost-effective structural components that are highly desirable. The effects of two surface treatment methods (i.e., “sandblasting” and “sandblasting with resin coating” denoted as hybrid approach) on the ultimate load-bearing capacity as well as the average shear and tensile strengths of single-lap MB-FML ABJs were investigated. Subsequently, the optimal overlap length of the SLJ was numerically established. The distribution of the stresses in the adhesive layers along and across the overlap length and width, respectively, was evaluated for SLJs with different overlap lengths. The important observations made in this study are highlighted as follows:

(1)The simple and cost-effective hybrid surface treatment method adopted in this study was demonstrated to be more effective in enhancing the response of the single-lap MB-FML ABJs than surface preparation by sandblasting. The joint prepared with the hybrid surface preparation technique resulted in a 15.6% and 15.7% improvement in average load capacity and shear strength, respectively, compared to the joints prepared with the conventional sandblasting surface preparation method. Moreover, the failure mechanism of SLJs transitioned from the adhesion (or interfacial) mode to a mixture of adhesion and cohesion mode by using the more effective surface preparation technique.
(2)The robust nonlinear FE framework constructed in LS-DYNA environment produced results with excellent correlation with the experimental results with a maximum error margin of 2.5% in predicting the joint capacity. It is believed that the developed model can be confidently used to assess the complex stress distribution along the adhesive layers in SLJs under various loading conditions.
(3)The ultimate capacity of the single-lap MB-FML ABJs could be enhanced by increasing the overlap length to a certain extent, beyond which no significant changes were observed.
(4)Increasing the adhesive thickness led to a greater joint capacity and improved the average shear and tensile strengths of the joints. The enhancement continued up to a certain adhesive thickness, beyond which a slight decrease in the capacity was observed.
